# Sin3A recruits Tet1 to the PAH1 domain via a highly conserved Sin3-Interaction Domain

**DOI:** 10.1038/s41598-018-32942-w

**Published:** 2018-10-02

**Authors:** Aditya Chandru, Neil Bate, Geerten W. Vuister, Shaun M. Cowley

**Affiliations:** 1 Department of Molecular and Cell Biology, University of Leicester, Lancaster Road, Leicester, LE1 7RH United Kingdom; 2 Leicester Institute of Structural and Chemical Biology, Leicester, United Kingdom

## Abstract

The Sin3A complex acts as a transcriptional hub, integrating the function of diverse transcription factors with histone modifying enzymes, notably, histone deacetylases (HDAC) 1 and 2. The Sin3A protein sits at the centre of the complex, mediating multiple simultaneous protein-protein interactions via its four paired-amphipathic helix (PAH) domains (PAH1-4). The PAH domains contain a conserved four helical bundle, generating a hydrophobic cleft into which the single-helix of a Sin3-interaction domain (SID) is able to insert and bind with high affinity. Although they share a similar mode of interaction, the SIDs of different repressor proteins bind to only one of four potential PAH domains, due to the specific combination of hydrophobic residues at the interface. Here we report the identification of a highly conserved SID in the 5-methylcytosine dioxygenase, Tet1 (Tet1-SID), which interacts directly with the PAH1 domain of Sin3A. Using a combination of NMR spectroscopy and homology modelling we present a model of the PAH1/Tet1-SID complex, which binds in a Type-II orientation similar to Sap25. Mutagenesis of key residues show that the 11-amino acid Tet1-SID is necessary and sufficient for the interaction with Sin3A and is absolutely required for Tet1 to repress transcription in cells.

## Introduction

The Sin3A protein is the central component of the multi-protein Sin3A complex, which along with its core components, histone deacetylase 1 and 2 (HDAC1/2), regulates chromatin structure and gene expression in all eukaryotic cells^1^. The complex lacks intrinsic DNA binding activity and is therefore recruited to specific loci by transcription factors, including Mxd1, PLZF and HBP1^2,3^. These factors ‘plug-in’ to the Sin3A complex via interactions mediated by four conserved paired amphipathic helix (PAH) domains (PAH1-4). PAH domains consist of a left-handed four helix bundle, forming a hydrophobic cleft into which a single helix from the transcription factor binds using specific hydrophobic and charged interactions^4,5^. While structurally similar, PAH domains have an intrinsic specificity for their binding partners. The repressor protein, Mxd1, for example, will bind tightly to Sin3A-PAH2, but not PAH1, 3 or 4^6,7^. This modular array of protein docking domains enables Sin3A to form a hub of transcription factors and chromatin modifying activities in the cell. Although HDAC1/2 form the core catalytic engine of the complex, Sin3A has also been associated with other enzymatic activities, including the O-linked N-acetylglucosamine transferase (OGT^8^) and helicases of the Swi/Snf chromatin remodelling complex^9^. Sin3A has also been found to interact with the 5-methylcytosine (5mC) dioxygenase, Ten-eleven translocation 1 (Tet1)^10,11^ although thus far its mode of interaction is unknown.

DNA methylation is a key epigenetic regulator of chromatin structure and gene expression whose pattern is maintained through cell division by DNA methyl transferase 1 (Dnmt1)^12^. Through a series of iterative oxidative steps, Tet1 (and its sister proteins Tet2 and Tet3) are able to partially convert 5mC to 5-hydroxymethylcytosine (5hmC), 5-formylcytosine (5fC) and finally 5-carboxylcytosine (5caC), thus initiating DNA demethylation, either by passive dilution during replication or enzymatically via the base excision repair pathway^13,14^. The association of Sin3A/HDAC1 with DNA demethylation activity, which at face value are opposing epigenetic modifications, may have an important role to play in stem cells. Williams *et al*.^11^, showed that Sin3A and Tet1 co-occupy many of the same target genes in embryonic stem (ES) cells. Moreover, knockdown of Tet1 causes a loss of Sin3A recruitment, suggesting that Tet1 recruits the Sin3A complex to DNA^11^. Here, we have identified a highly conserved Tet1 Sin3A interaction domain (Tet1-SID) which interacts directly with the PAH1 domain of Sin3A. Using a combination of NMR spectroscopy and homology modelling we present a model of the PAH1/Tet1-SID complex, which binds in a similar orientation (type-II) to Sap25. Mutagenesis of key residues show that the 11-amino acid Tet1-SID is necessary and sufficient for the interaction with Sin3A and are absolutely required for Tet1 to repress transcription in cells.

## Results

### Tet1 and Tet3 contain a highly conserved Sin3 interaction domain (SID)

**Figure 1:**
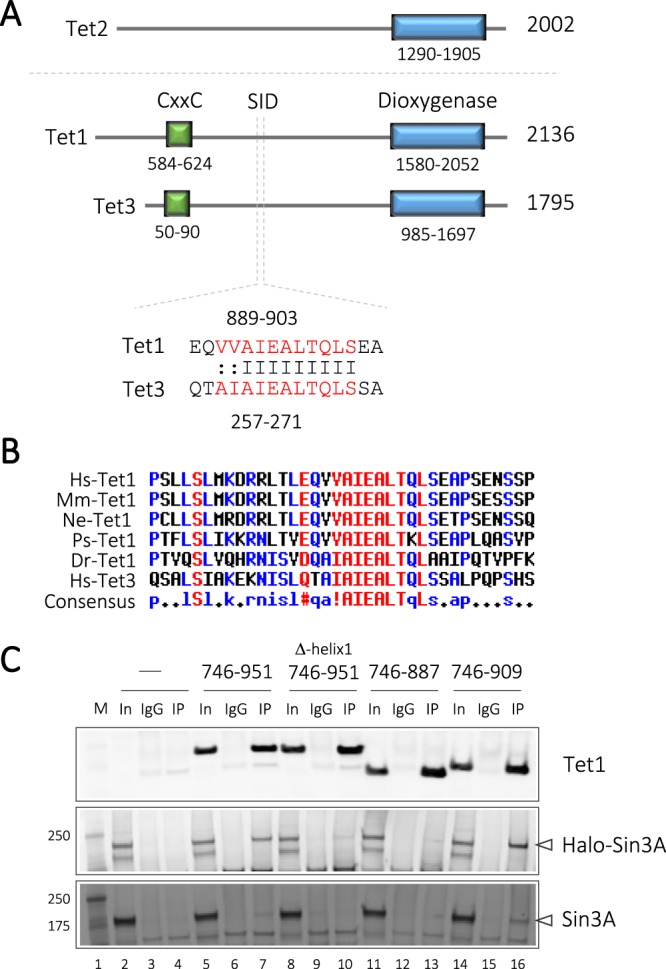
Identification of a conserved Sin3 Interaction Domain (SID) in Tet1 and Tet3. (**A**) Schematic diagram of Tet1, 2 and 3 showing the presence of a putative helical SID in Tet1/3, but not Tet2. (**B**) The putative Tet1-SID is highly conserved across multiple species; Hs- *Homo sapiens*, Mm – *Mus musculus*, Ne - *Notamacropus eugenii* (Wallaby), Ps - *Pelodiscus sinensis* (Chinese soft shell turtle), Dr - *Danio rerio*. (**C**) Co-immunoprecipitation followed by western blotting reveals the requirement of the Tet1-SID for association with exogenous and endogenous Sin3A. This image is cropped, the uncropped version of the blot is shown in Supplementary Fig. S5A.

Previously, Williams *et al*.^11^, and Deplus *et al*.^10^, showed that Tet1 and Tet3 interact with Sin3A, whereas Tet2 does not. Crucially, the rationale for this differential behaviour was not identified. We therefore postulated that a yet unidentified characteristic region would be present in Tet1 and Tet3, but not in Tet2. Thus, we began by aligning Tet1, 2 and 3 and searching for conserved regions between the three proteins using the online alignment tool, Multalin^15^. All three proteins contain a conserved dioxygenase domain which catalyses the conversion of 5mC to 5hmC (Fig. 1A). Tet1 and 3 also contain an N-terminal Zn-finger CxxC (ZnF-CxxC) domain which binds to unmethylated CpGs within the genome. Using a combination of protein-protein sequence alignment and secondary structure prediction (Jpred^16^) we identified a putative Sin3 interaction domain (SID) with high similarity between Tet1 (889–903) and Tet3 (257–271), which was predicted to have a helical structure. This region was particularly outstanding since there is little or no predicted secondary structure outside of the already known dioxygenase and ZnF-CxxC domains of Tet1 (see Supplementary Figs S1 and S2 for full alignments). A comparison of this region among Tet1 proteins from other species reveals it to be highly conserved in human, mouse, wallaby, turtle, and zebra-fish (Fig. 1B). Significantly, the putative SID is absent from Tet2 or its dimeric partner, IDAX, thus potentially accounting for the differential Sin3A binding characteristics of Tet1/3 and Tet2.

To determine if the putative SID was required for the Sin3A/Tet1 interaction, we performed co-immunoprecipitation experiments with myc-tagged Sin3A and fragments of Tet1 in 293 T cells. These experiments show that a region of Tet1 (746–951) containing the putative SID binds to Sin3A, whereas a C-terminal truncation of this region (746–887), or a deletion of the predicted SID helix (746–951 Δhelix1) fails to interact (Fig. 1C; compare lanes 7, 10 and 13). We were also able to detect a small amount of endogenous Sin3A binding (lanes 7 and 16). We have therefore identified a conserved Tet1-Sin3A interaction domain (Tet1-SID) which is necessary for binding to Sin3A.

**Figure 2:**
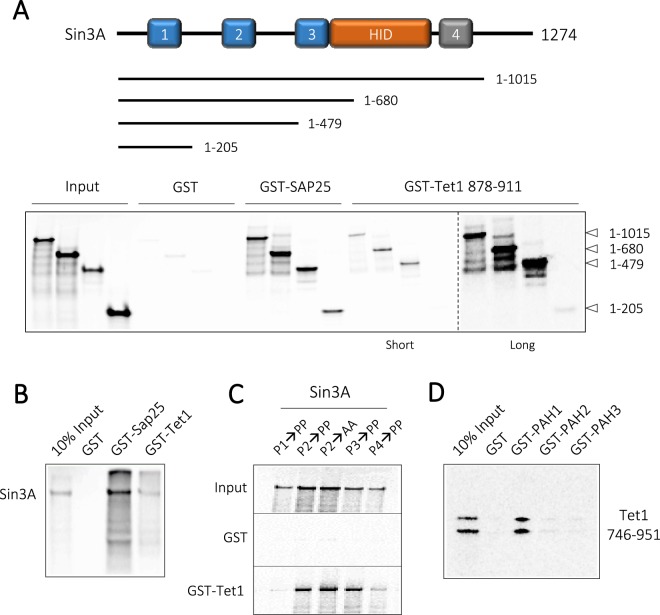
The Tet1-SID binds to the PAH1 domain of Sin3A. (**A**) Schematic diagram showing the boundaries of Sin3A deletion constructs and relevant domains, PAH domains 1, 2, 3 and 4 are indicated by boxes. PAH4 is shaded in grey as it is likely non-functional. HID – HDAC interaction domain. Lower panel, GST-pulldown with the SID domains of Sap25 and Tet1 using ^35^S-Met using the indicated Sin3A truncations. Short and long exposures of the gel were taken to visualize the binding of Tet1 to PAH1 as indicated. (**B**) GST-pulldown experiment using the indicated GST-fusion proteins with ^35^S-Met labelled full-length Sin3A. (**C**) GST-pulldown with GST-Tet1 and full-length ^35^S-Met labelled Sin3A. Mutations in individual PAH domains (P1–P4) to either Proline (PP) or Alanine (AA) are indicated. This image is cropped, the uncropped version of the gel is shown in Supplementary Fig. S5B. (**D**) GST-pulldown with GST-PAH domains (1–3) and Tet1 746–951 labelled with ^35^S-Met.

Having identified the Tet1-SID, a series of Sin3A C-terminal truncations were used to map the Tet1 binding region within Sin3A (Fig. 2A). In a series of pulldown experiments we compared the binding of GST-Tet1(878–911) with GST-Sap25, a protein known to bind specifically to the PAH1 domain^17^. Both Tet1 and Sap25 bound all of the Sin3A fragments tested (Fig. 2A – lower panel), suggesting that Tet1 binds to PAH1, albeit less efficiently than Sap25, since a long exposure was required to visualize binding to the Sin3A 1–205 fragment (Fig. 2A,B and Supplementary Fig. S3). It is interesting to note that Tet1 binds better to a Sin3A fragment containing both PAH1 and PAH2 domains (1–479) than PAH1 alone (1–205), suggesting that there might be additional regions that contribute to binding or that intermolecular PAH1/PAH2 interactions affect PAH1 affinity for Tet1. Using a series of Sin3A mutants with proline mutations in each of the four PAH domains^7^, we found that only mutations in PAH1 disrupted Tet1 binding (Fig. 2C). Finally, we confirmed the binding of the Tet1-SID to PAH1 by using the isolated PAH1, PAH2 or PAH3 domains, and only GST-PAH1 was capable of binding Tet1 746–951 (Fig. 2D). Collectively, these data indicate the Tet1-SID binds to PAH1 domain of Sin3A.

### The Tet1-SID is an amphipathic helix with critical hydrophobic residues

**Figure 3:**
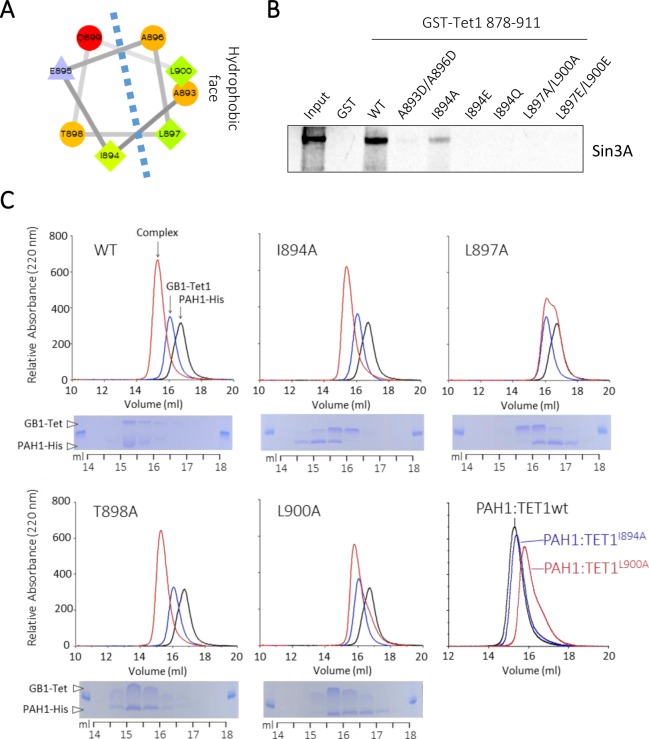
The Tet1-SID forms an amphipathic helix with key hydrophobic residues required for interaction with Sin3A. (**A**) Helical wheel of the Tet1-SID residues 893–900 demonstrates hydrophilic and hydrophobic faces. (**B**) GST pulldown using GST-Tet1 878–911 wild-type and mutations, as indicated, with ^35^S-Met labelled Sin3A. This image is cropped, the uncropped version of the gel is shown in Supplementary Fig. S5C. (**C**) Column fractionation of purified GB1-Tet1 and PAH1-His (mixed at a 1:1 ratio) demonstrates their ability to form a binary-complex in solution, which is dependent on the presence of key hydrophobic residues.

Jpred analysis of full-length Tet1 suggests the region comprising the Tet1-SID forms an α−helix. Application of a helical wheel analysis showed that A893, I894, A896, L897 and L900 form a hydrophobic face capable of interacting with PAH1 (Fig. 3A). Moreover, these residues are 100% conserved across different species and with Tet3 (Fig. 1B). The presence of a pair of short (A893/A896) and long (L897/L900) hydrophobic side-chains is reminiscent of the lock-and-key interaction observed in the Mxd1/PAH2 complex^7^. We generated a series of point mutants to probe the relative contributions of specific residues to the Tet1-Sin3A interaction. The requirement for the long side-chains in the Tet1-SID domain was confirmed, since an L897A/L900A mutation completely abolished the interaction with Sin3A in a GST-pulldown experiment (Fig. 3B). The hydrophobic nature of the interaction was also confirmed, as the introduction of charged residues to the Tet1-SID (A893D/A896D) also abolished Sin3A binding. Similarly, substitution of hydrophobic residue at I894 (I894A) reduced binding, but was tolerated, whereas introduction of a charged residue (I894E) was not. To further examine the contribution of individual hydrophobic residues to complex formation, we expressed and purified GB1-Tet1(878–911) and His-PAH1 domains individually, mixed them at a 1:1 ratio and then tested their ability to form a binary-complex using analytical gel filtration chromatography. Formation of a PAH1:Tet1-SID complex was assessed by an increase in mass and an earlier elution time (left shift) relative to the individual components (Fig. 3C). An L897A substitution completely eliminated binding (no size shift), while L900A also markedly reduced complex formation, identifying this pair of Leucine residues as critical components of the Tet1-SID. Consistent with the pull-down experiments, the I894A mutation could also be tolerated. An overlap of the WT/I894A/L900A data (bottom right panel) reveals their relative contribution to binding and indicates that I894 has a more modest contribution to binding in comparison to L900 or L897. Mutation of T898 to Ala had no effect on binding, suggesting that it does not form part of the interaction interface or that any effects of the Threonine Cγ-methyl group can easily be accommodated by the Alanine Cβ-methyl.

**Figure 4:**
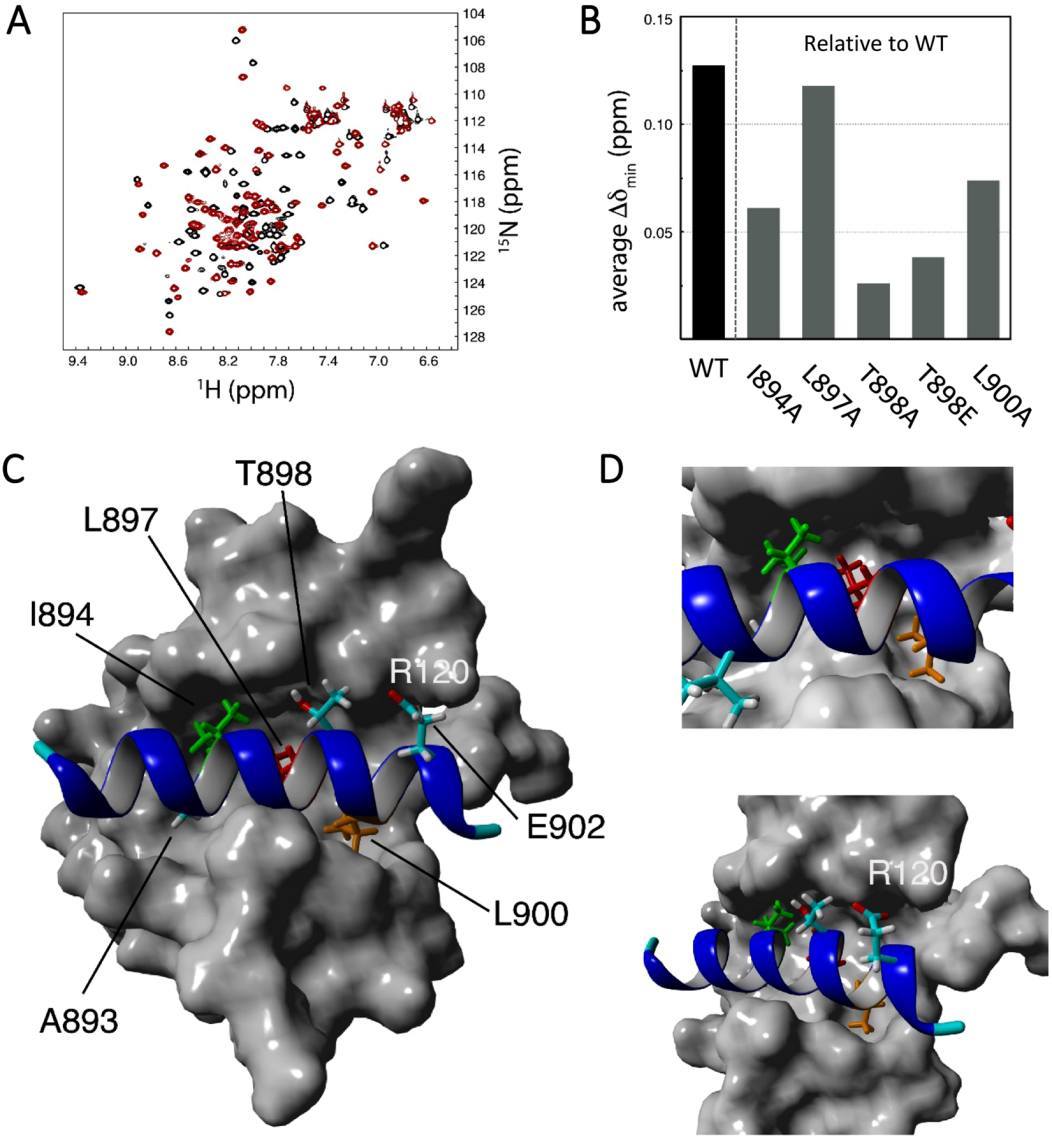
NMR and structural modelling of the PAH1:Tet1 complex. (**A**) Overlay of the ^15^N-HSQC spectra of apo-PAH1 (black) and wild-type PAH1:Tet1 complex (red). All NMR data was recorded at 303 K. (**B**) Results of minimal chemical shift mapping of various PAH1-Tet1 complexes. Value for the WT complex (coloured black) is computed relative to the apo state and establishes the magnitude of the effect. Values for mutant complexes are computed relative to the WT-complex and thus are a measure for the importance of the mutated residue for complex formation. (**C**) Model of the PAH1:Tet1 complex derived from the PAH1:Sap25-SID NMR ensemble (see methods). PAH1 is shown in surface representation; the Tet1 peptide in ribbon presentation with crucial residues as sticks: A893, I894 (green), L897 (red), T898, L900 (orange), and E902. The latter is potentially capable of an electrostatic interaction with PAH1-R120. (**D**) Close-ups of the PAH1:Tet1 complex; crucial residues shown and highlighted as in (**C**).

The Tet1-SID and PAH1 constructs were also used to express and label purified proteins for analysis by high-resolution heteronuclear NMR spectroscopy. Consistent with previous studies^18,19^, the ^15^N-HSQC spectra showed that PAH1 is structured in the absence of a partner (Fig. 4A). Addition of the Tet1-SID(878–911) at a 1:1 stoichiometric ratio causes significant changes in the cross peak positions, indicative of a conformational change in PAH1 upon binding Tet1 (Fig. 4A). The peaks respond to the addition of Tet1 according to a so-called slow-exchange regime, as exemplified by the appearance of both apo and bound cross peaks at sub-stoichiometric ratio, which signifies a significant binding affinity estimated to be K_d_ < 100 nM. Conversely, ^15^N-HSQC spectra of the free Tet1-SID show it to be largely unfolded, with a major shift towards a folded stretch of ~15 amino acids upon addition of PAH1, with most of these in an α-helical conformation (Supplementary Fig. S6).

Chemical shift changes are a sensitive probe to establish protein-protein binding, able to detect both major and more subtle changes in the structure resulting from the interaction, as well as changes in dynamics. By comparing the spectra of apo-PAH1 complexed with the Tet1-SID, or its mutants (Supplementary Fig. S4), a good assessment of the relative contributions of Tet1-SID residues to the interaction can be established. To quantify the observed spectral changes and assess the effects of each mutation, we performed a minimal-shift analysis^20^ and computed the average minimal shift, Δδ_min_, using the sets of all matched peaks between the various ^15^N-HSQC spectra (see methods). As expected on the basis of the visual comparison, the largest Δδ_min_ is obtained from the comparison of the spectra of the apo-protein and the wild-type complex (Δδ_min_ = 0.127). Consistent with the crucial role of L897 in formation of the complex, the ^15^N-HSQC spectrum of the Tet1-SID L897A mutant complex showed the largest difference relative to that of the WT complex (Δδ_min_ = 0.118). Indeed, of all mutant complexes, the ^15^N-HSQC spectrum of the L897A mutant most resembles that of the apo-protein (Δδ_min_ = 0.061, Supplementary Fig. S4C), indicating that the L897A mutation causes a near complete loss of Tet1 binding. Also consistent with the pulldown and gel filtration experiments, the ^15^N-HSQC spectrum of the L900A complex revealed a more modest change relative to the WT complex (Δδ_min_ = 0.074), while the changes in the ^15^N-HSQC spectrum of I894A (Δδ_min_ = 0.061) appear smaller compared to the effects caused by either the L897A or L900A mutations. The ^15^N-HSQC spectrum of T898A most resembled that of the WT complex (Δδ_min_ = 0.026), whereas the complex with the T898E mutation only displays minor changes relative to WT (Δδ_min_ = 0.038), indicating that the PAH1 domain is able to accommodate the bulky and charged glutamate sidechain with modest disruption. Collectively, this suggests that the importance of the contributions to the PAH1:Tet1-SID interface decreases as L897 > L900 > I894 > T898.

The structures of the various PAH domains in complex with their target SIDs have shown the structural requirement for a helical LxxL-motif^19^, as also observed for Tet1. Surprisingly however, two orientations, denoted as type-I or type-II, of the SID when bound to the PAH1 domain have been observed^19^, as exemplified by the PAH1:Sap25-SID (PDB code 2RMS^19^) and PAH1:REST (PDB code 2CZY^18^) structures. The transcriptional repressor REST binds PAH1 with the N-terminal portion of the SID pointing directly into the hydrophobic cleft (type-I), while the Sap25-SID is rotated by 180° relative to REST (type-II). Using these two structures as a starting point, we generated two models for the PAH1:Tet1 complex (see methods) and used the relative contribution of individual residues (Figs 3B,C and 4B) as a guide to the selection of the most likely version (Fig. 4C and Supplementary Fig. S7). In both potential orientations, L897 anchors Tet1 into the PAH1 domain, consistent with its crucial role in complex formation. Since L900 is more important for binding relative to I894, this supports a model based upon Sap25 (type-II). Furthermore, only the model based upon the type-II orientation will accommodate a T898E mutation (Supplementary Fig. S7B), which does not affect PAH1:Tet1 binding (Fig. 4B). Overall, the Sap25-based PAH1:Tet1 model displays good geometry with plausible packing of the two proteins resulting in 753 Å^2^ buried surface area, whereas the PAH1:REST (type-I) derived model yields a compromised helix and a much less convincing interface (Supplementary Fig. S7B). Additional electrostatic interactions can potentially be formed in the PAH1:Sap25 derived model between PAH1 R120 and Tet1 E902, in line with previous studies on PAH domain mediated recognition that showed a role for electrostatic interactions in addition to the core LxxL Sin3 interaction motif^21^.

### Critical hydrophobic resides in the Tet1-SID are required for Sin3-dependent transcriptional repression

**Figure 5:**
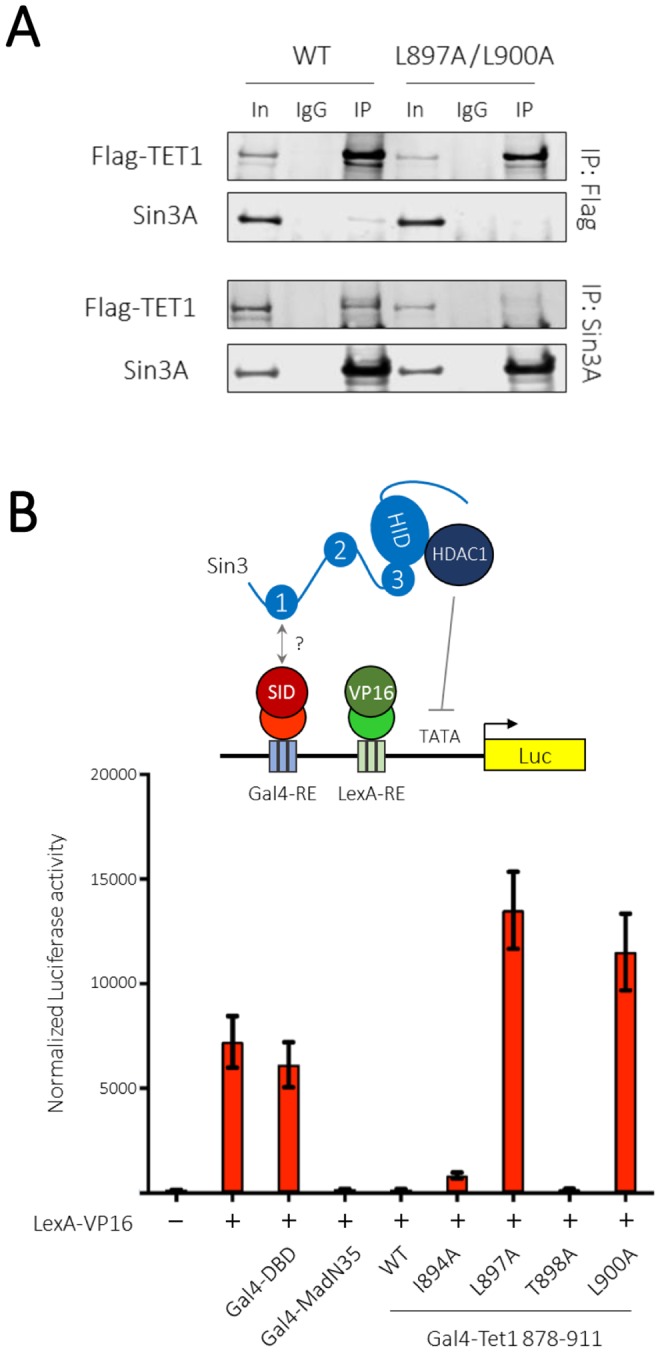
Tet1 L897 and L900 are key residues for interaction with Sin3A in cells. (**A**) Co-immunoprecipitation followed by western blotting reveals the requirement of L897 and L900 for the interaction with endogenous Sin3A in mouse ES cells. This image is cropped, the uncropped version of the blot is shown in Supplementary Fig. S5D. (**B**) Transcription of a luciferase reporter assay is driven by a simple TATA based promoter enhanced by addition of a LexA-VP16 fusion protein. Transcription can be repressed by co-expression of either the Mxd1-SID (MadN35), or Tet1-SID fused to a Gal4 DNA binding domain (DBD). Repression is dependent upon the interaction with Sin3A, since mutation of the Tet1-SID causes loss of repression.

Having defined L897 and L900 as critical hydrophobic residues required for the interaction with PAH1 *in vitro*, we next assessed their influence on Tet1 activity in cells. We used wild-type and Tet1-SID mutant (L897A/L900A) constructs to generate ES cell lines stably expressing full-length flag-tagged Tet1 (Fig. 5A). Co-IP using Flag-antisera revealed that wild-type Tet1 was able to associate with endogenous Sin3A, but that mutation of the Tet1-SID disrupts this interaction. This result was confirmed by performing the reciprocal co-IP using endogenous Sin3A as the bait (Fig. 5A, bottom panel). The results confirm the *in vivo* requirement for L897 and L900 as critical residues of the Tet1-SID, required for interaction with Sin3A. The recruitment of Sin3A/HDAC1 to genomic loci is thought to be necessary for the activity of many different transcriptional repressors. Indeed, the SIDs of Mxd1 and Sap25 have been demonstrated to be necessary and sufficient to repress transcription in reporter gene assays^7,17,22^. We therefore fused the Tet1-SID (878–911) to the Gal4-DNA binding domain and measured its ability to modulate the transcription in a reporter assay (Fig. 5B). As a control we used the well described 35 amino acid Mxd1-SID (Gal4-MadN35), a potent transcriptional repressor^7,22^. Gal4-MadN35, but not the Gal4-DBD alone, was able to significantly repress transcriptional levels. Remarkably, Gal4-Tet1(878–911) inhibited transcription to the same degree as Gal4-MadN35. However, mutation of either L897A, or L900A completely abolished repression, strongly suggesting that the ability to suppress transcription is dependent on Sin3A recruitment, consistent with the activity of many other SID containing repressors. In agreement with its limited involvement in mediating the PAH1-Tet1 interaction (Figs 3 and 4), mutation of T898A did not affect activity. Interestingly, I894A, a residue which contributes to PAH1 binding towards the end of the hydrophobic pocket (Fig. 4C), is still able to inhibit transcription, although not quite to the same degree as the wild-type Tet1-SID, suggesting that it is of intermediate importance.

## Discussion

We have identified a highly conserved region of Tet1 which is necessary and sufficient for its interaction with Sin3A. Similar to the Sin3 interaction domain (SID) of other transcriptional repressors (Mxd1^4,5^, REST^18^, HBP^23^, etc.), the Tet1-SID consists of a single helix with an arrangement of specific hydrophobic residues which form a lock and key interaction with one of four potential Sin3A PAH domains (PAH1-4). Mutation of critical hydrophobic residues within the TET1-SID (L897A/L900A) was sufficient to completely abolish binding of the two proteins in cells (Fig. 5A). Zhong *et al*., recently showed binding of Sin3A to the C-terminal region of Tet1^24^, however we find no interaction with this region. Mapping experiments using Sin3A truncation and point-mutants revealed that the Tet1-SID binds specifically to the PAH1 domain. Interestingly, binding was more pronounced in a Sin3A construct containing both PAH1 and PAH2 (Sin3A 1–479) than PAH1 alone (1–205), suggesting that there may be cooperativity between these two adjacent PAH domains, a phenomenon also observed for Mxd1-SID/PAH2^7^. The isolated PAH1 domain, which displays a classical four helical-bundle topology in the absence of a SID^19^, is able to form a complex with purified Tet1-SID(878–911), resulting in a general conformational change in each domain (Fig. 4A and Supplementary Fig. S6). Tet1 joins the repressors REST^18^ and PLZF^25,26^, and the chromatin associated proteins, Sap25^17^ and HCF-1^27^, as PAH1 interacting proteins. The helical SID domains that bind to PAH1 can do so in either a type-I (REST) or type-II (Sap25) orientation which differ in a 180° rotation of the ligand relative to the PAH1 domain^19^. Mutagenesis experiments (Figs 3B,C and 4B) and homology modelling (Fig. 4), imply that the Tet1-SID binds in the type-II orientation as also observed in the PAH1:Sap25 complex. Since multiple proteins are able to bind PAH1, this suggests that there may be a variety of Sin3A complexes *in vivo*, each with a unique arrangement of associated factors, determined by the relative binding affinities and availability of individual proteins in the cell. In competition experiments with Sap25-SID and Tet1-SID (Fig S3), binding of Sap25 to PAH1 appeared to be stronger than Tet1, suggesting that a hierarchy of PAH1-binding proteins might exist. However, this assumes that all components have similar stoichiometries. This was recently addressed by Streubel *et al*.^28^ who used co-IP of endogenous Sin3A coupled with quantitative mass-spectrometry. They showed that the majority of Sin3A associated proteins, including Tet1, are sub-stoichiometric members of the complex, suggesting that Sin3A (or PAH1) may not be a limiting factor in complex assembly in cells.

It is interesting to speculate on the functional consequences of the Sin3A:Tet1 interaction. At face value, the amalgamation of histone deacetylation (classically repressive) and a 5mC hydroxylase (which potentially leads to DNA demethylation), ought to have opposing effects on gene regulation. However, genome-wide ChIP experiments for HDAC1 have shown that it maps preferentially to transcriptional start sites (TSS) of active genes, questioning the conventional wisdom of HDACs exclusively leading to transcriptional repression^29^. Tet1 also maps to the TSS of active genes, as does its product, 5hmC^11^. Indeed, Williams *et al*.^11^, showed that Tet1 and Sin3A co-occupy many genes in ES cells and that Tet1 was required for the recruitment of Sin3A to target genes. We show here that the Tet1-SID is a robust transcriptional repression domain, equivalent in potency to the Mxd1-SID, and that its activity is dependent upon its interaction with Sin3A (Fig. 5B). Tet1 is still able to modulate gene expression even in DNA methyltransferase triple KO cells, i.e. cells lacking any 5mC. This argues in favour of Tet1 being able to function as a classical DNA-binding transcriptional repressor via its ZnF-CxxC domain, recruiting Sin3A/HDAC1 to target genes and modifying rates of transcription, in addition to its well described role in 5mC hydroxylation. Interestingly, both Tet1 and Tet3 which both bind to DNA directly have a conserved SID, whereas Tet2, which binds to DNA via heterodimeric partner IDAX does not, hinting at a relationship between direct DNA binding and Sin3A recruitment.

In conclusion, we have discovered a novel and highly conserved SID domain within Tet1 which is necessary and sufficient for its interaction with Sin3A. While our manuscript was under revision, Zhu *et al*.^30^, reported the binding site of Tet1 to the Sin3A PAH1 domain, and found that the Tet1/PAH1 association was required for ES cell pluripotency. Sin3A helps recruit Tet1 to genes such as the Nodal antagonist, Lefty1, maintaining its transcriptional activity and thus preventing commitment towards a mesendodermal lineage^30^. These data underscore the importance of this ‘odd couple’ of chromatin modifying activities and hints at additional roles in the epigenetic regulation of gene expression.

## Methods

Protocols, materials and constructs used in the study will be made available upon request.

### Cell culture and transfection experiments

293 T cells were maintained in Dulbecco modified Eagle medium supplemented with 10% foetal calf serum, penicillin-streptomycin, and glutamine. Cells were transiently transfected with plasmid constructs using Lipofectamine 2000 (ThermoFisher) according to the manufacturer’s instructions. For transient repression assays, cells were plated on gelatinized 24-well tissue culture plates and then transfected with Gal4.LexA.TATA.Luc (500 ng), β-actin-LacZ control plasmid (150 ng), Gal4.Mxd1 or Gal4.Tet1-SID (100 ng), and LexA.VP16 (100 ng). A constant amount of DNA was maintained in each sample by the addition of an empty vector. At 48 h after transfection, cells were harvested and assayed for luciferase (Biovision) and β-galactosidase activities using standard protocols.

### Co-Immunoprecipitation and western blot assays

For co-immunoprecipitation (co-IP) experiments were performed as described previously^31^, 500 μg of whole cell extract, prepared from transfected 293 T cells, was incubated overnight at 4 °C with antibody-coated (anti-Flag, or anti-Sin3A) protein-G sepharose beads in IP buffer (250 mM NaCl, 10% Glycerol, 0.5% IEGPAL, 50 mM Tris at pH7.5). Following incubation, beads were washed four times in IP buffer. Captured proteins were resolved by SDS/PAGE, transferred to a nitrocellulose membrane and then probed with anti-Flag (Sigma, M2 monoclonal), anti-Sin3A (Ab129087) or anti-Halo Tag (Promega G921A) antisera and then scanned using a LI-COR Odyssey scanner.

### GST pulldown assays

These were based on the protocols used previously in Cowley *et a*l.^7^. Briefly, recombinant cDNAs for mSin3A, Tet1 and their mutant derivatives were transcribed and translated *in vitro* using a TNT reticulocyte lysate kit (Promega) in the presence of [^35^S]-methionine according to the manufacturer’s instructions. GST-Tet, GST-Sap25, GST-PAH1-3 constructs and mutant derivatives were expressed in *Escherichia coli* DH5α and purified by using glutathione-Sepharose (GE). mSin3A proteins labelled with [^35^S]-methionine were incubated with the GST fusions proteins bound to Sepharose beads in a buffer containing 150 mM NaCl, 20 mM Tris (pH 8.0), 1 mM EDTA, and 0.5% IGEPAL CA-630 for 3 h and then washed three times in the same buffer. Bound proteins were analysed by SDS-PAGE and then visualized with a phosphorImager.

### Production of purified PAH1 and Sin3 interaction domains (SID)

Histidine tagged Sin3A-PAH1 115–212 (PAH1), SAP25 126–186 (Sap25-SID) and TET1 878–911 (TET1-SID) were produced using BL21 Star (DE3) *E*. *coli*. The latter two proteins contained an additional GB1 solubility tag. PAH1 was ^15^N labelled using M9 minimal media containing (^15^N) NH_4_Cl. Following IPTG induction and cellular lysis, affinity chromatography was used to elute the tagged proteins from the greater lysate. In the case of the TET1/Sap25 proteins, these were dialyzed into a 20 mM Tris-Cl pH8, 50 mM NaCl and 1 mM DTT solution using a 3500 MW membrane. The dialyzed TET1/Sap25 proteins were subsequently purified and concentrated using anion exchange chromatography. Tag removal for NMR was conducted as needed, following the dialysis, using a histidine tagged TEV protease. This permitted the separation of both the tag and the TEV from the untagged protein solution using the same nickel column.

### NMR methods

The ^15^N-^1^H HSQC NMR spectra of the various PAH1-Tet1 complexes (1:1) were recorded at 303 K on Bruker 600 MHz AVIII, 600 MHz AVIII HD, or 800 MHz AVII spectrometers equipped with CryoProbes. Samples contained 150 µM ^15^N-labelled PAH1(115–212) and 150 µM unlabelled Tet1(878–911) in 20 mM Tris-HCl pH 7.4, 50 mM NaCl and 1 mM DTT, 95:5 v/v % H_2_O/D_2_O. NMR data processing was performed using TOPSPIN v3.1 and analysis using the CcpNmr Analysis^32^ and AnalysisAssign^33^ programmes. The spectra were peak-picked automatically at ~1.4 the noise level of the spectrum, followed by manual curation of spurious peaks. All peaks from the Asn, Gln NH_2_ sidechains were excluded from the analysis. The average minimal shift values over all peaks between two spectra, Δδ_min_, were used to assess the effects of mutations as proposed by Bate *et al*.^34^. It served to establish the differential effects of the mutation, i.e. the extent to which a specific residue contributes to the interaction. The Δδ_min_ value for each spectral comparison was defined as the summed difference over all matched peaks divided by the number of peaks. Peaks were matched between the peak lists of the relevant spectra using a minimal distance algorithm (typically 70–75 peaks). Peak distances Δδ were calculated using the common 7:1 ^1^H:^15^N weighting. The whole procedure was implemented in an in-house python script using the SciPy library and the AnalysisAssign API to access the NMR data.

### Molecular modelling

The first members of the PAH1:Sap25-SID NMR ensemble (PDB code 2RMS^19^) and the PAH1:REST NMR ensemble (PDB code 2CZY^18^) were used as the starting point for the molecular modelling of the PAH1-Tet1 complex using the program YASARA-Structure^35^ (www.yasara.org). The two structures displayed a 0.9 Å backbone RMSD over the four helices. Both the Sap25-based model and the REST-based model were generated using identical procedures. First, the peptide was mutated *in-silico* to the homologous Tet1 residues and any major steric clashes manually resolved by sampling residue-specific preferred sidechain rotamer conformations. Next, the structure of the Tet1 peptide was optimised by a short MD simulation in H_2_O using the YAMBER4 force field^36^ and periodic boundary conditions, keeping the PAH1 part fully fixed. Next, another short MD simulation was performed, with residues in the PAH1-Tet1 interface additionally allowed to adjust. The final Sap25-based model displayed plausible overall conformational characteristics, together with a well-defined PAH1-Tet1 interface (Fig. 4C,D) covering 753 Å^2^. In contrast, the Tet1 peptide in the REST-based model displayed a somewhat distorted helical conformation, presumably from a non-optimal fit. Crucially, in the REST-based model T898 directly faces the hydrophobic binding pocket of the PAH1 domain, whereas it is significantly solvent exposed in the Sap25-based model.

## Electronic supplementary material

Supplementary Figures
