# Supplementary material for: Sin3A recruits Tet1 to the PAH1 domain via a highly conserved Sin3-Interaction Domain
Source: Sci Rep. 2018 Oct 2;8:14689. doi: 10.1038/s41598-018-32942-w (PMC6168491; doi:10.1038/s41598-018-32942-w)
Supplement: Supplementary file 1 — Supplementary Figures [file 41598_2018_32942_MOESM1_ESM.pdf]

# Sin3A recruits Tet1 to the PAH1 domain via a highly conserved Sin3-Interaction Domain

Aditya Chandru[1], Neil Bate[1,2], Geerten W. Vuister[1,2] and Shaun M. Cowley[1,*]

[1] Department of Molecular and Cell Biology, University of Leicester, Lancaster Road, Leicester, LE1 7RH, United Kingdom
[2] Leicester Institute of Structural and Chemical Biology

Supplementary Figures

# Fig S1

## Protein alignment of full-length Tet1 and Tet3

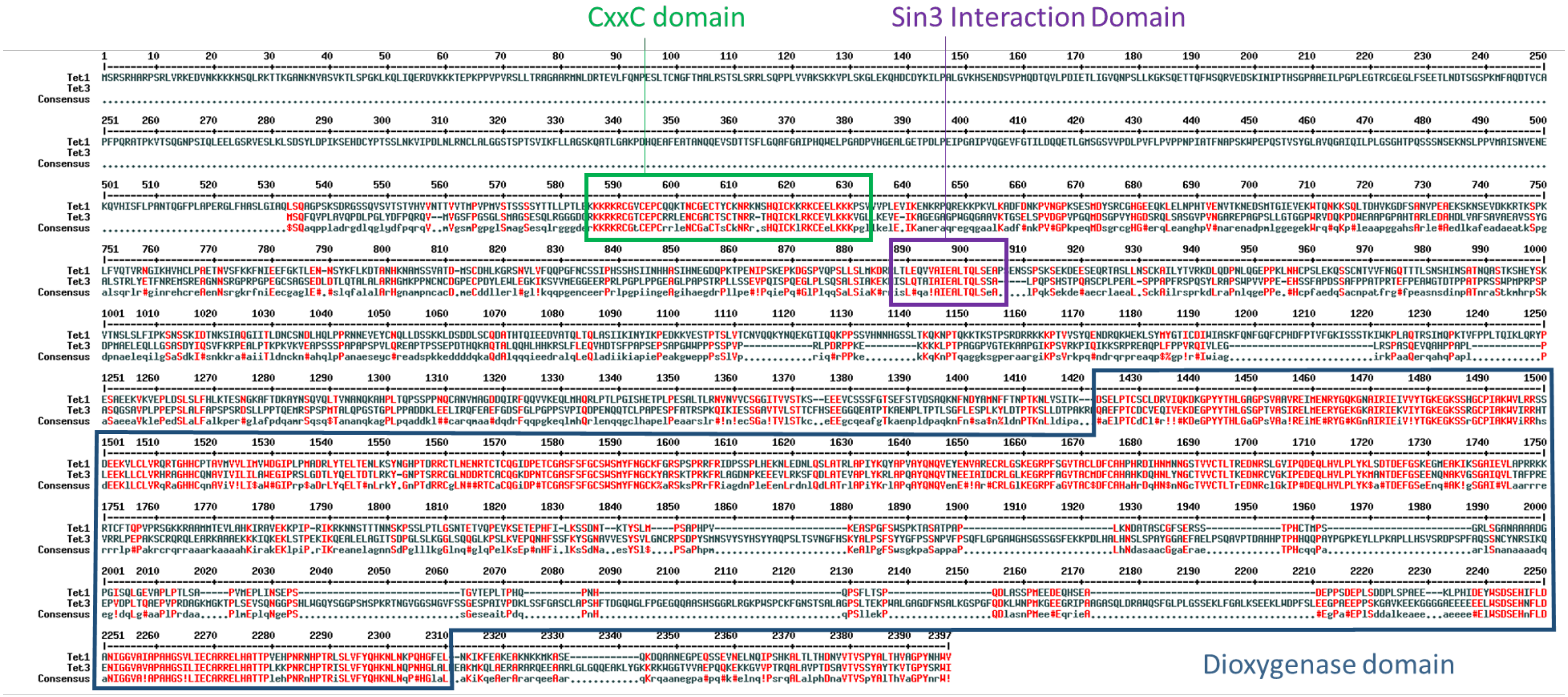


Supplementary Figure S1 – Multalin (Corpet, F. Multiple sequence alignment with hierarchical clustering. *Nucleic Acids Res* **16**, 10881-10890 (1988)), was used to align full-length Tet1 and Tet3 proteins. Red = identical, blue = similar residues. The position of CxxC, Sin3 interaction domain (SID) and dioxygenase domains are indicated by the coloured boxes.

Fig S2

# Protein alignment of full-length Tet1, Tet2 and Tet3

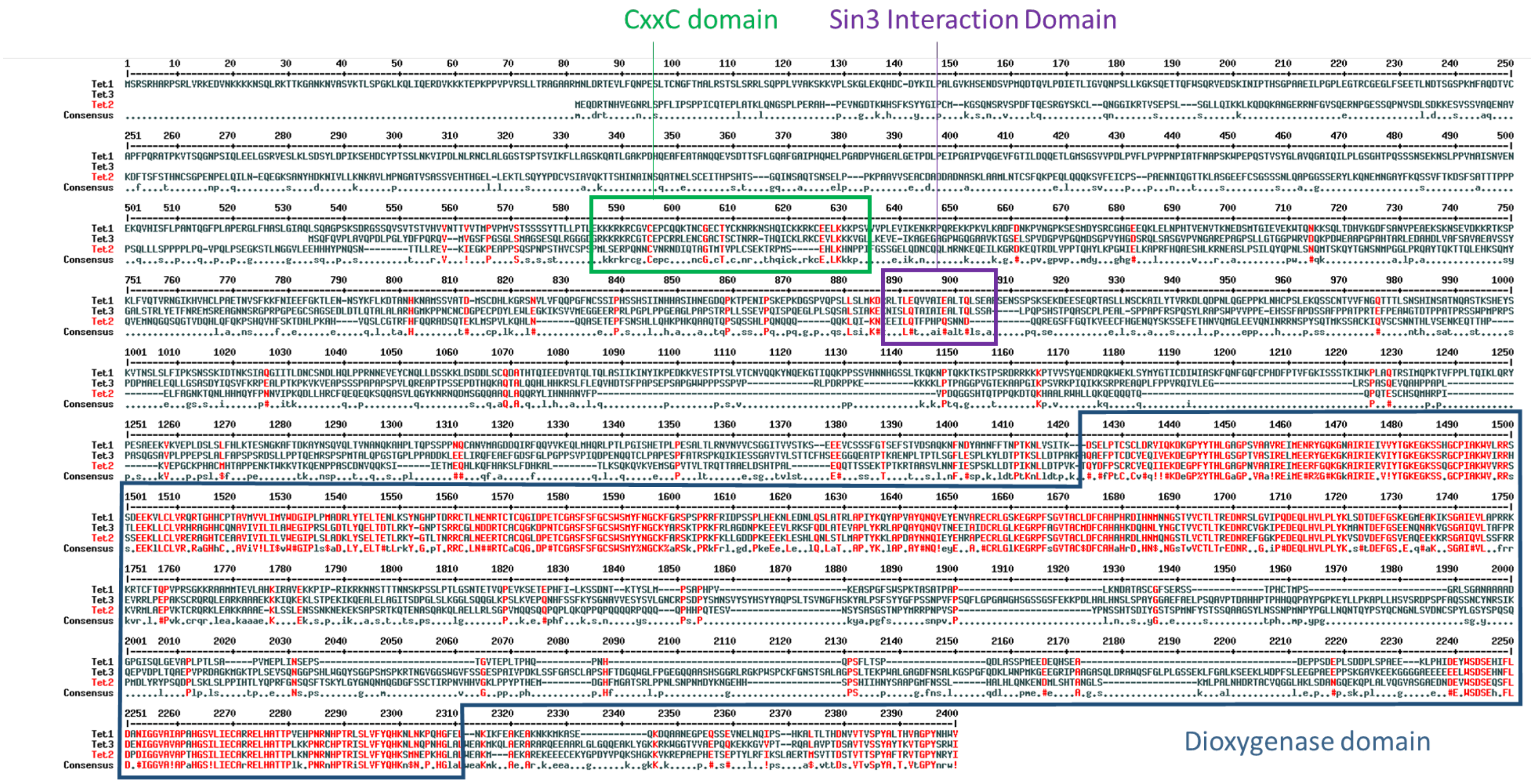


Supplementary Figure S2 – Multalin (Corpet, F. Multiple sequence alignment with hierarchical clustering. *Nucleic Acids Res* **16**, 10881-10890 (1988)), was used to align full-length Tet1, Tet2 and Tet3 proteins. Red = identical, blue = similar residues. The position of CxxC, Sin3 interaction domain (SID) and dioxygenase domains are indicated by the coloured boxes.

Fig S3

# SAP25 out competes TET1 for PAH1 binding

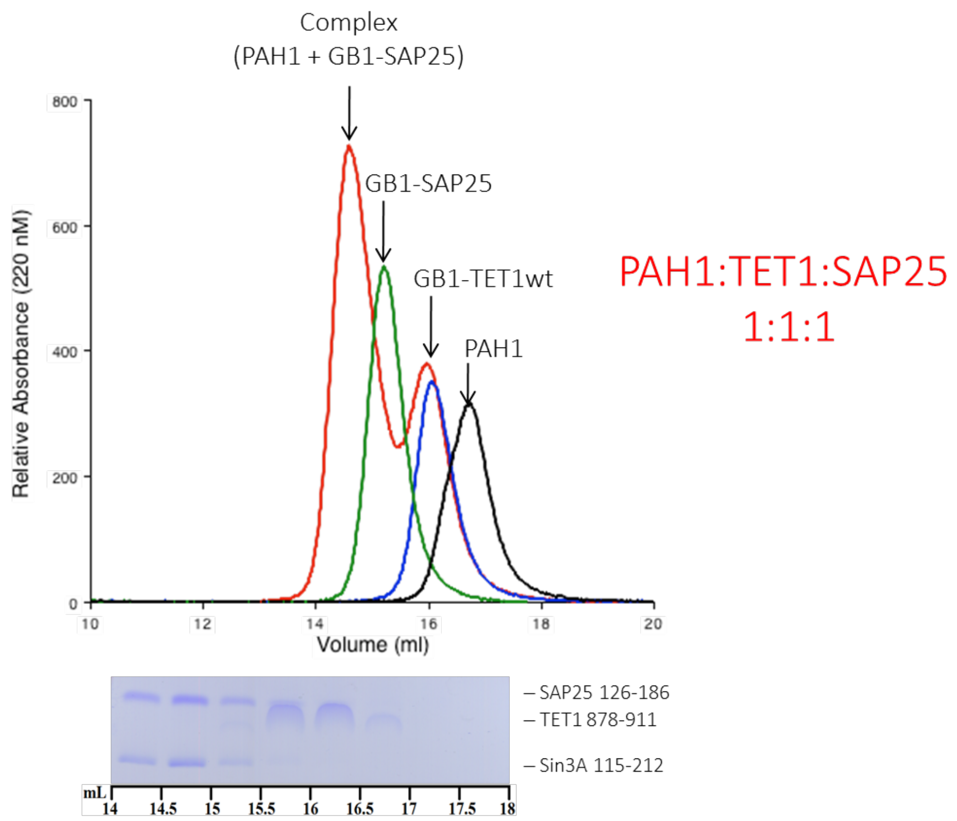


Coomasie scan of 0.5mL fractions from 14mL to 18mL
of SAP25 126-186, TET1 878-911 and Sin3A 115-212

Gel Filtration: 20 mM TRIS-Cl pH 7.4, 50 mM NaCl, 1 mM DTT

Supplementary Figure S3 – Column fractionation of purified GB1-Tet1, GB1-SAP25 and PAH1-His (mixed at a 1:1:1 ratio) demonstrates that the Sap25-SID outcompetes the Tet1-SID for binding to Sin3A-PAH1 domain.

# Fig S4

Supplementary Figure S4. NMR analysis of Tet1 mutants.

A) Overlay of the $^{15}N$-HSQC spectra of apo-PAH1 (red) and wildtype PAH1:Tet1 complex (black). (Figure identical to Fig 4A).

B) Overlay of the $^{15}N$-HSQC spectra of wildtype PAH1:Tet1 complex (black) and PAH1:Tet1-I894A complex (red).

C) Overlay of the $^{15}N$-HSQC spectra of wildtype PAH1:Tet1 complex (black) and PAH1:Tet1-L897A complex (red).

D) Overlay of the $^{15}N$-HSQC spectra of wildtype PAH1:Tet1 complex (black) and PAH1:Tet1-L900A complex (red).

E) Overlay of the $^{15}N$-HSQC spectra of wildtype PAH1:Tet1 complex (black) and PAH1:Tet1-T898A complex (red).

F) Overlay of the $^{15}N$-HSQC spectra of wildtype PAH1:Tet1 complex (black) and PAH1:Tet1-T898E complex (red).

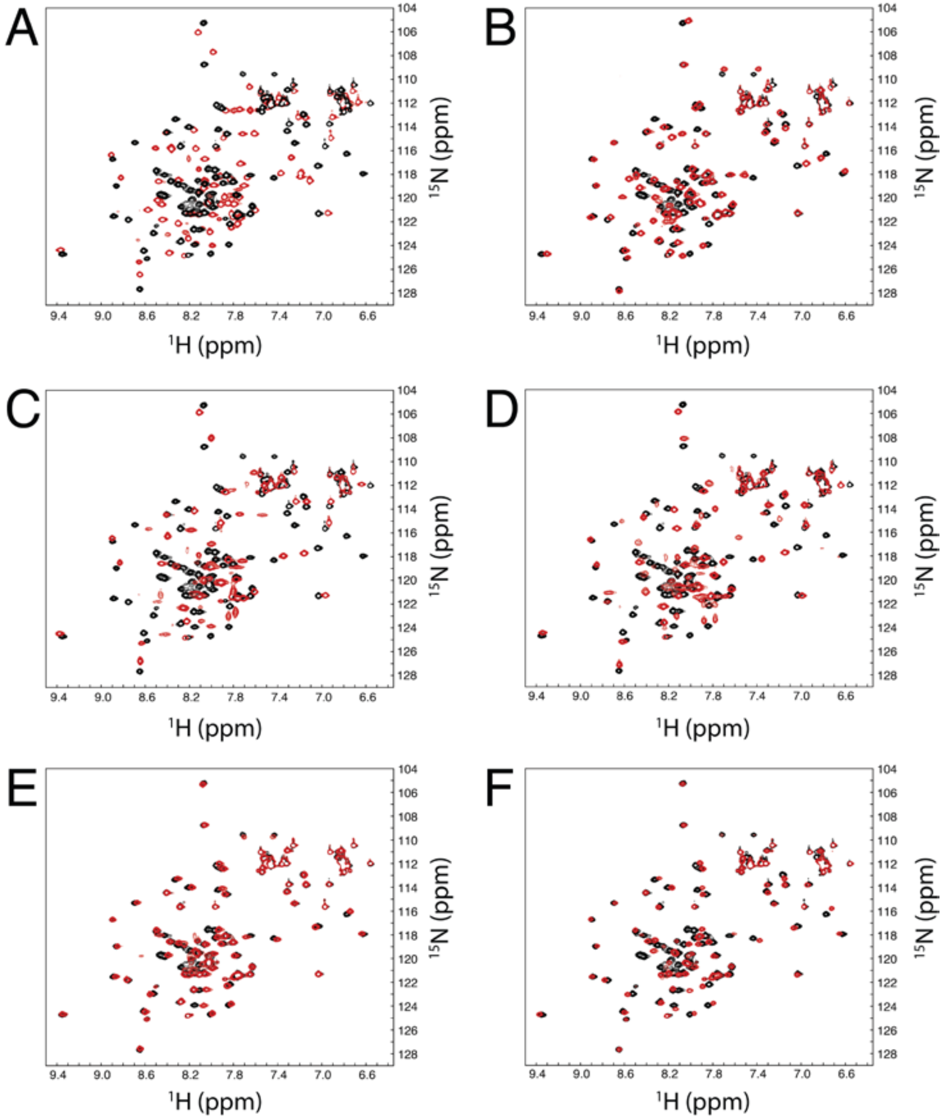

# Fig S5

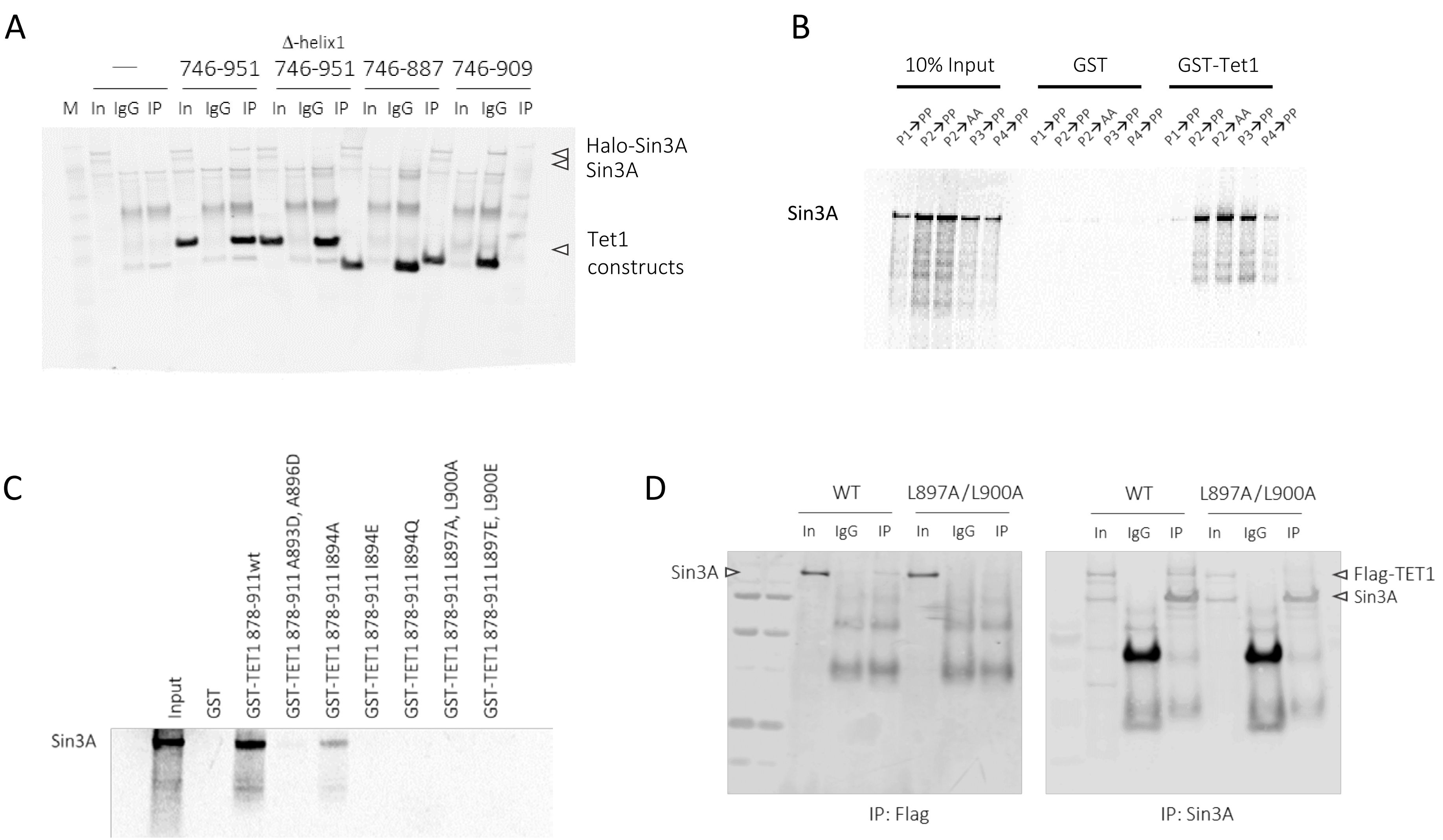


Supplementary Figure S5 – Uncropped blots A) Fig. 1C; B) Fig. 2C; C) Fig. 3B and D) Fig. 5A

# Fig S6

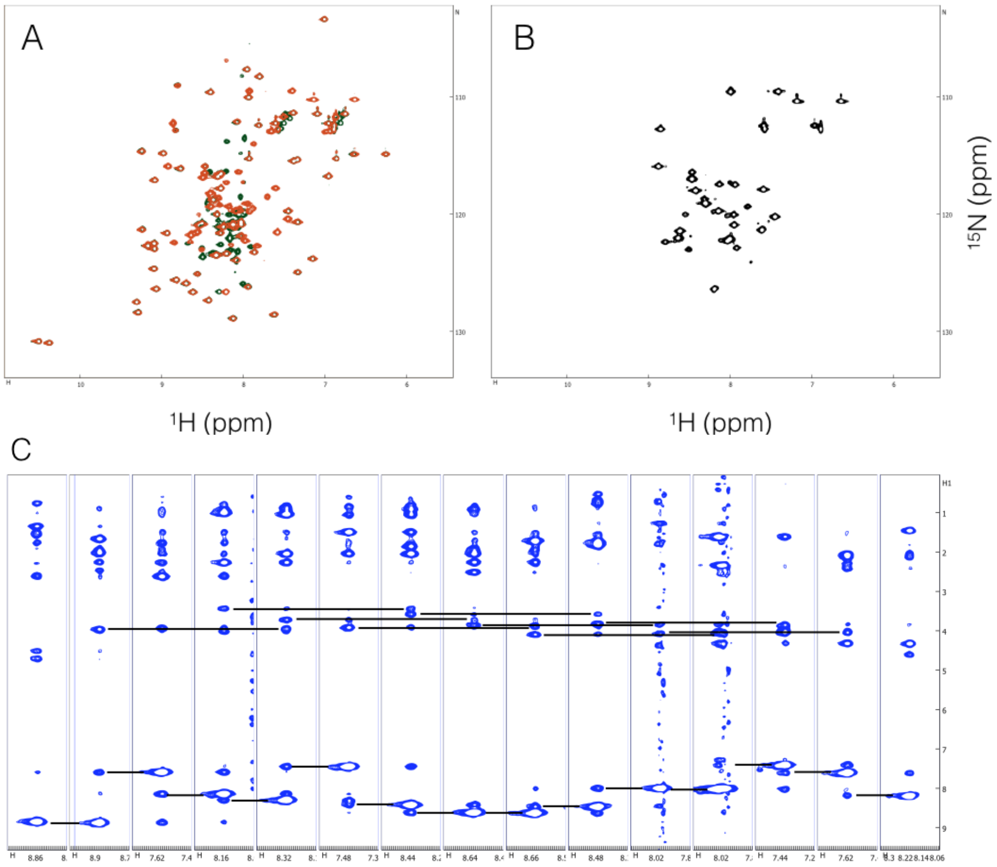


Supplementary Figure S6. NMR of TET1(870-911) A) Overlay of the $^{15}$N-HSQC spectra of $^{15}$N-labelled GB1-TET1 (green) and $^{15}$N-labelled GB1-TET1 in complex with unlabelled PAH1 (orange). The cross peaks of GB1 tag superimpose exactly. In contrast, the visible isolated green peaks are characteristic of the unfolded TET1 residues. The peaks of the folded TET1 residues are to some extent indistinguishable from the GB1 peaks. B) $^{15}$N-HSQC spectrum of $^{15}$N-labelled TET1 in complex with unlabelled PAH1, as obtained from after cleavage of the GB1 tag. The cross peaks of the folded TET1 residues are now clearly visible. C) Sequential $^1$H strips of the $^{15}$N-NOESY-HSQC spectrum of $^{15}$N-labelled TET1 in complex with unlabelled PAH1, taken at the ($^1$H,$^{15}$N) resonance frequencies of the peaks observed in B). The sequential $d_{NN}$(i-1,i) and selected $d_{\alpha N}$(i, i+3) Noe's, all characteristic of $\alpha$-helical conformation, are indicated. Also note the many Noe's in the methyl region of the spectrum, as expected from the hydrophobic TET1-PAH1 interface, including the LxxL PAH-binding motif of TET1.

Fig S7

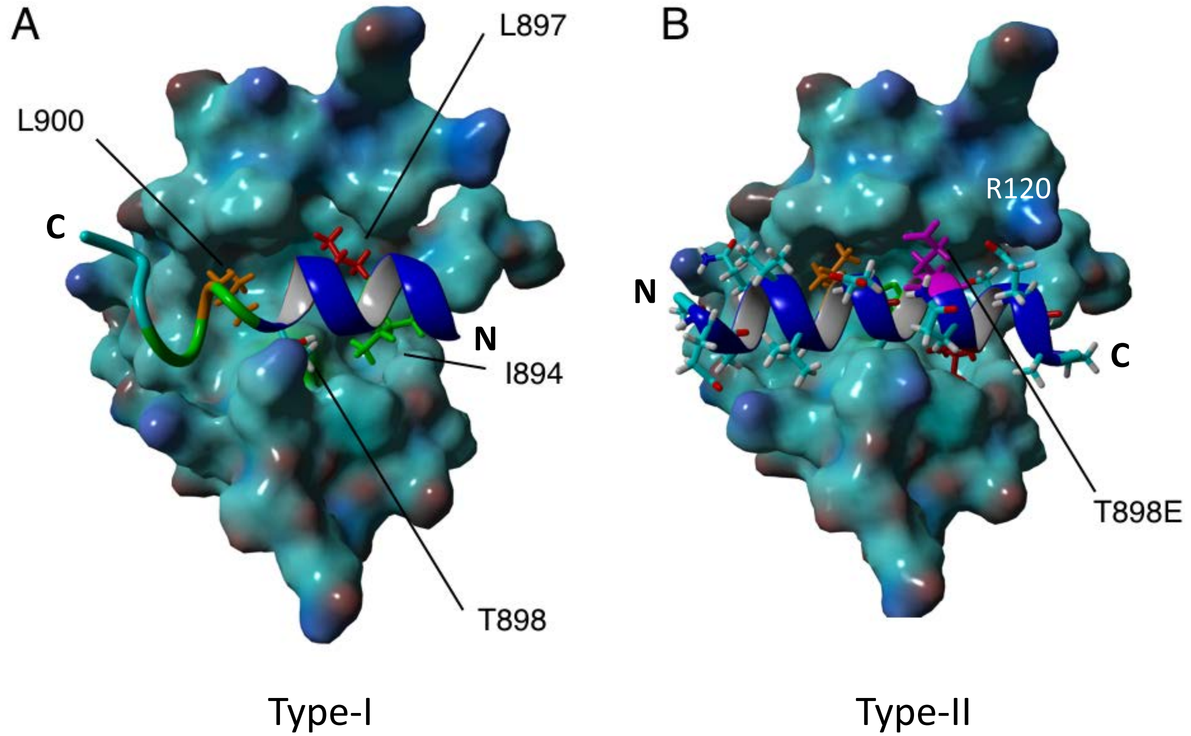


Supplementary Figure S7. A) Model of the PAH1:Tet1 complex derived from the PAH1:REST-SID (type-I) NMR ensemble (see methods). PAH1 is shown in surface representation coloured according to electrostatic potential in an orientation identical to Fig. 4C. Consequently, the Tet1 peptide in ribbon presentation with crucial residues as sticks: I894 (green), L897 (red), T898, L900 (orange), and is rotated 180° degrees relative to Figs 4C and B). B) Model of the PAH1:Tet1 complex derived from the PAH1:Sap25-SID (Type-II) NMR ensemble (as in Fig. 4C); the T989E mutation is shown in purple.
